# The stress distribution around a thick-walled cylinder by a proposed constitutive model for rocks

**DOI:** 10.1371/journal.pone.0307878

**Published:** 2024-08-15

**Authors:** Zhaofa Wu, Chengfeng Liu

**Affiliations:** 1 Shanxi Coal Transportation and Marketing Group Dongda Energy Co., Ltd., Shanxi, China; 2 Tiandi Technology Co., Ltd., Beijing, China; University of Education, PAKISTAN

## Abstract

To simulate the nonlinear stress-strain curve of rocks under static loads and contribute to the design and construction of rock engineering structures, a constitutive model has been proposed based on the elastic modulus E decreasing with the increase in longitudinal cracks. This constitutive equation offers numerous advantages, with the most noteworthy being that the simulation of stress-strain curves for rocks necessitates only three equations (Eqs 1–3) and four parameters (*A*, *k*_0_, *C* and *ε*_*s*_). Following this, we employ the constitutive equation to analyze the stress distribution around a thick-walled cylinder and explore the impact of its four parameters on the stress distribution surrounding the thick-walled cylinder. Parameter *A* primarily affects the range of the plastic zone and the magnitude of the maximum tangential stress; parameter *C* mainly influences the magnitude of the maximum tangential stress; parameter *ε*_*s*_ mainly affects the range of the plastic zone and the magnitude of the maximum tangential stress; parameter *k*_0_ primarily influences the magnitude of the maximum tangential stress. We got the similar results with Bray model, but distribution of stress around the tunnel are different present that the shape of stress-strain curves are different.

## 1. Introduction

**Fig 1:**
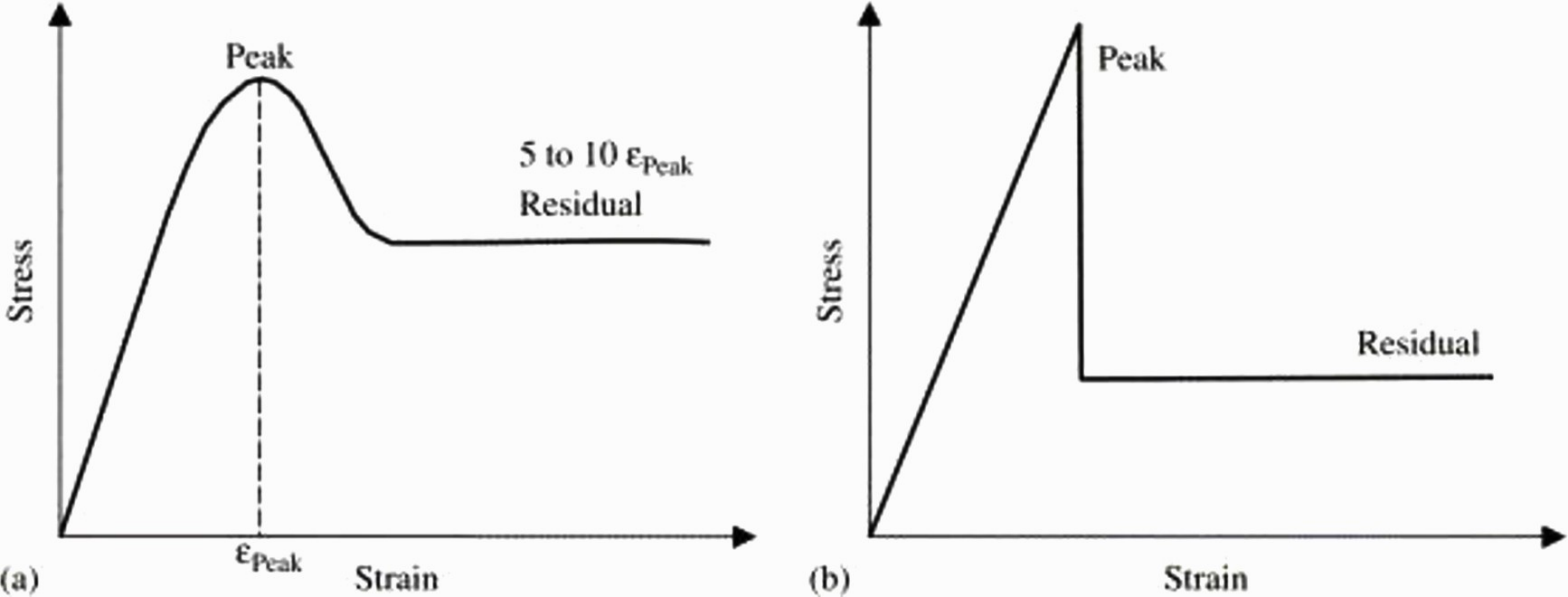
(a) Stress-strain curve of rock (b) Ideal stress-strain curve [5].

There are several failure criteria, such as the well-known Mohr-Coulomb (MC) and Hoek-Brown (HB) failure criteria, or criteria improved based on these two types of strength criteria. All proposed based on Fig 1B and widely used in rock engineering, such as foundations, slopes, tunnels, underground caves, galleries, and mining sites. Peak strength refers to the maximum strength a rock can endure, while residual strength denotes the stabilized strength following the peak, typically occurring within a strain range approximately 5–10 times that of the strain at peak strength. To simplify the study of rock strength, the real stress-strain curve of rocks (Fig 1A) is often presented in a simplified form, as depicted in Fig 1B. However, failure criteria based on Fig 1B cannot better simulate the real axial stress-strain curve of rocks (Fig 1A) and the lateral stress-strain curve of rocks, thus providing limited guidance and analysis for the design and construction of rock engineering structures.

Therefore, many researchers proposed constitutive models to simulate the nonlinear stress-strain curves of rocks [1–4, 6]. Currently, there are two mechanistic types of rock constitutive equations: one is elastoplastic constitutive equations, such as traditional ones based on Mohr-Coulomb (MC) and Hoek-Brown (HB) failure criteria [7–9]; the other is elastic constitutive equations proposed based on Hooke’s law [10, 11], such as the Fujii elastic constitutive equation. Compared to elastoplastic constitutive equations, elastic constitutive equations are relatively simpler, have fewer parameters, and therefore have more potential for practical applications [12].

In this paper, a constitutive model has been proposed based on the elastic modulus E decreasing with the increase in longitudinal cracks. Subsequently, this constitutive equation is employed to analyze the stress distribution around a thick-walled cylinder, investigating the impact of the parameters within the constitutive equation on the stress distribution surrounding the thick-walled cylinder. The proposed model is compared with the Bray model.

## 2. A constitutive model

**Fig 2:**
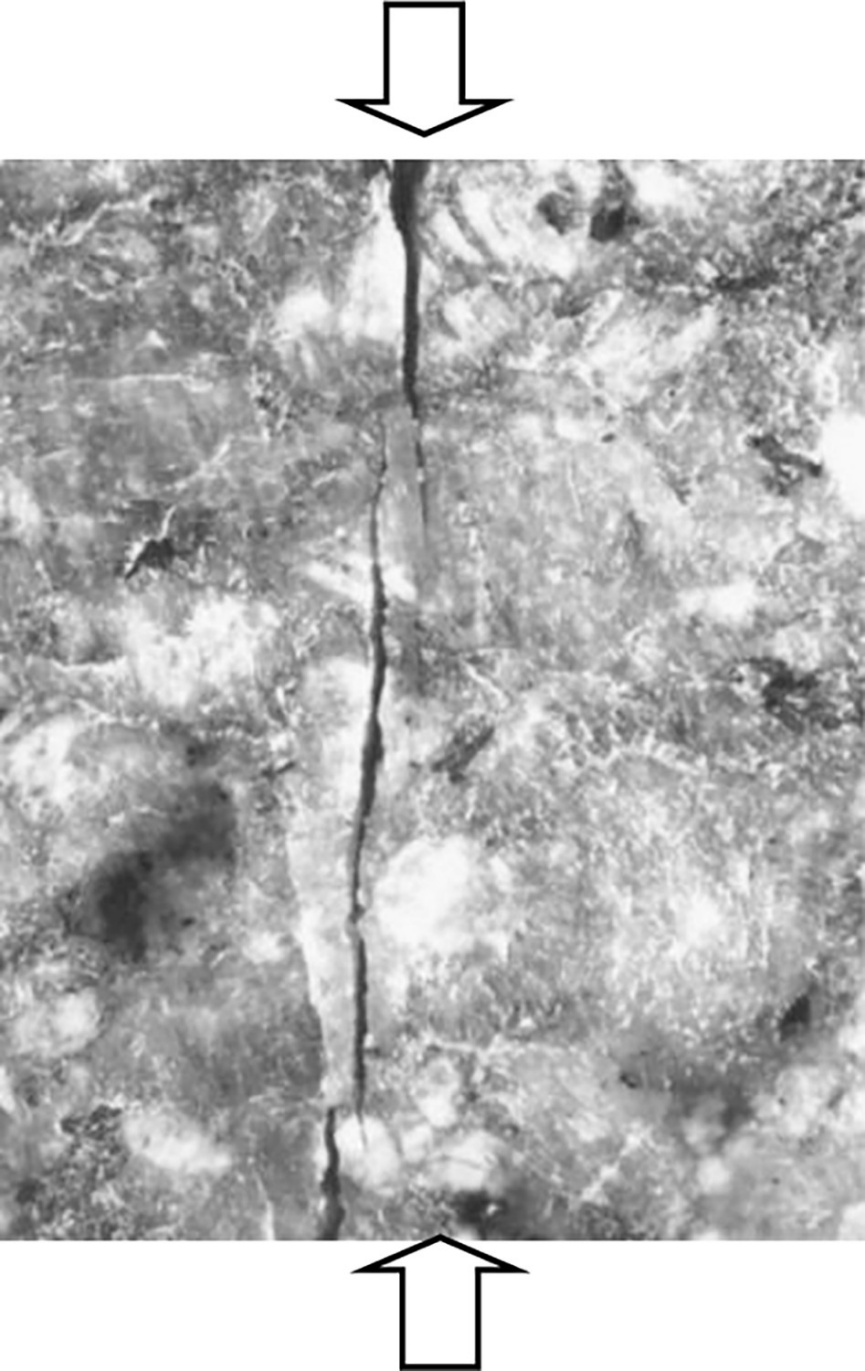
Uniaxial experimental model of rock [5] and [13]. With the increase of axial pressure, the formation and development of horizontal cracks lead to the decrease of horizontal elastic modulus.

A constitutive model has been proposed based on the observed trend of the elastic modulus E decreasing with the increase in longitudinal cracks, as illustrated in Fig 2,

$$\sigma_{1}=E'(\varepsilon_{1})\varepsilon_{1}+vE\varepsilon_{2}$$ (1)

$$\sigma_{2}=E'(\varepsilon_{2})\varepsilon_{2}+vE\varepsilon_{1}$$ (2)

$$E^{'}(\varepsilon )=\frac{\left(A-k_{0}A\right)}{\pi }[\frac{2\;\cot^{-1}\{k_{0}(\frac{\varepsilon }{\varepsilon_{s}}+5)\}}{\pi }]+k_{0}A$$ (3)

Where *A*, and *k*_0_ are constant, *E* are Young modulus, and *ν* is the Poisson’s ratio.

**Fig 3:**
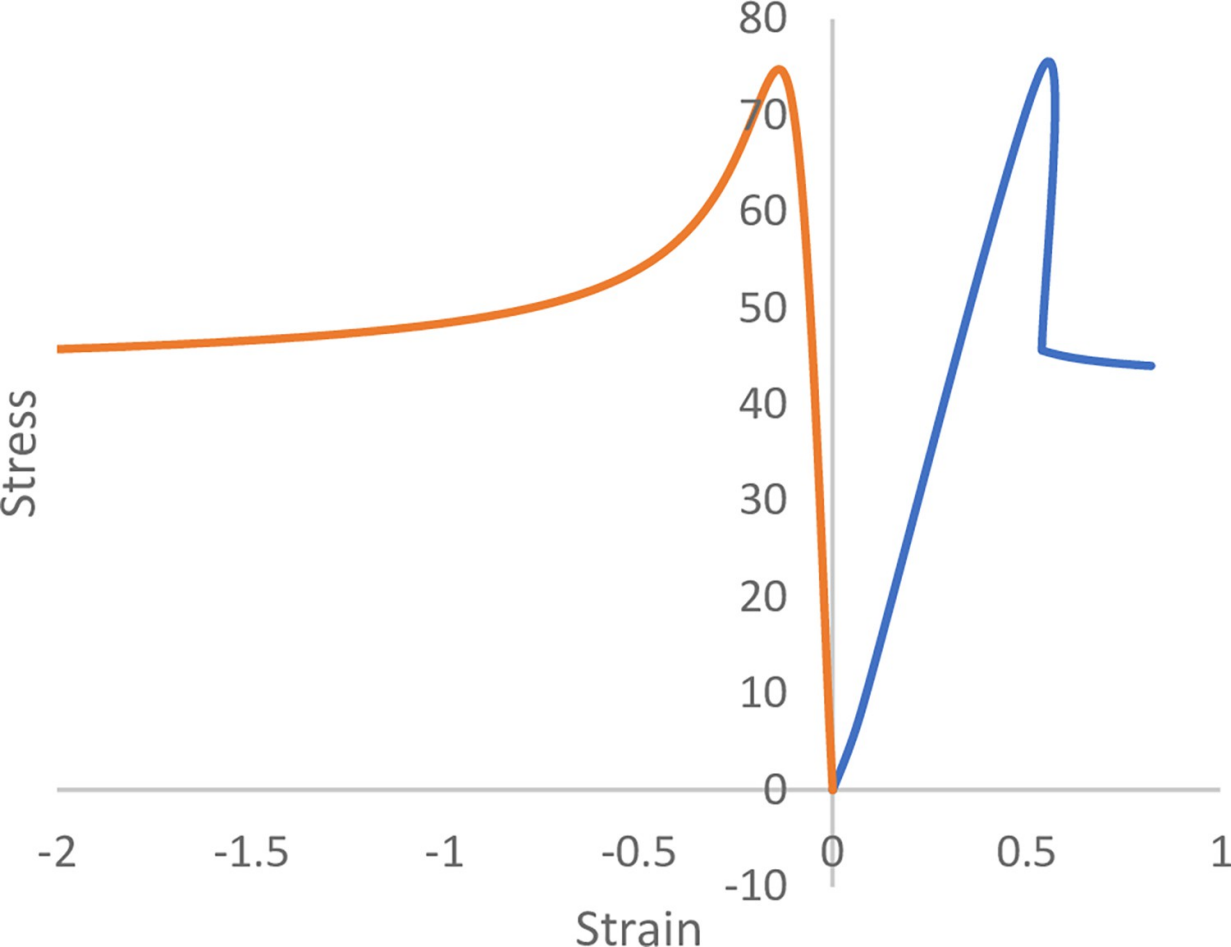
The constitutive model, parameters *A* = 15 GPa, *k*_0_ = 0.1, *C =* 1, and *ε*_s_ = -0.1.

From Fig 2, it can be observed that during the rock failure process, longitudinal cracks initiate and develop, ultimately leading to the fracture of the rock. Thus, the constitutive model proposes Eq (3) based on the decrease in elastic modulus in the longitudinal direction during the rock failure process. With the introduction of Eq (3), this constitutive equation allows for the derivation of the elastoplastic rock stress-strain curve (Fig 3). The most significant characteristic is that this constitutive equation requires only three equations (Eqs 1–3) and four parameters (*A*, *k*_0_, *C* and *ε*_*s*_).

**Fig 4:**
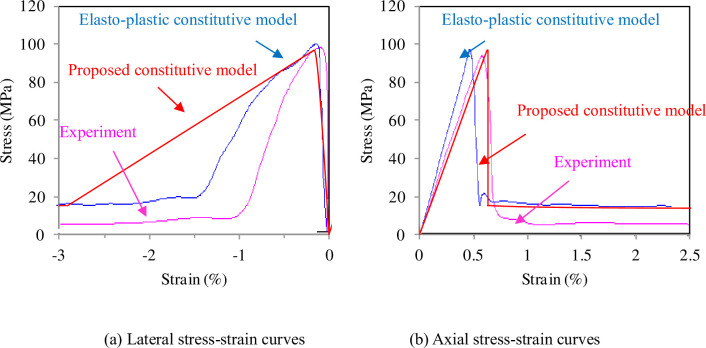
Stress-strain curves of sandstone under the uniaxial compression. (a) Lateral stress-strain curves. (b) Axial stress-strain curves.

The proposed simple model approximates the experimental results (Fig 4A and 4B) with only four parameters, *A =* 16.62 GPa, *k*_0_ *=* 0.18, *C =* 3.5, and *ε*_*s*_ *=* 0.002, and the results including Young’s modulus *E* = 23 GPa, *ν* = 0.135, friction angle *ϕ* = 49.36, cohesion *c*_0_ = 17.81 MPa, and four fitting parameters: *n* = 0.29, *m* = 0.1, *G* = 7.486, *H* = 28.351, are represented by the blue lines in Fig 4A and 4B.

## 3. Calculation of stress in thick-walled cylinder

### 3.1. Analysis of results

**Fig 5:**
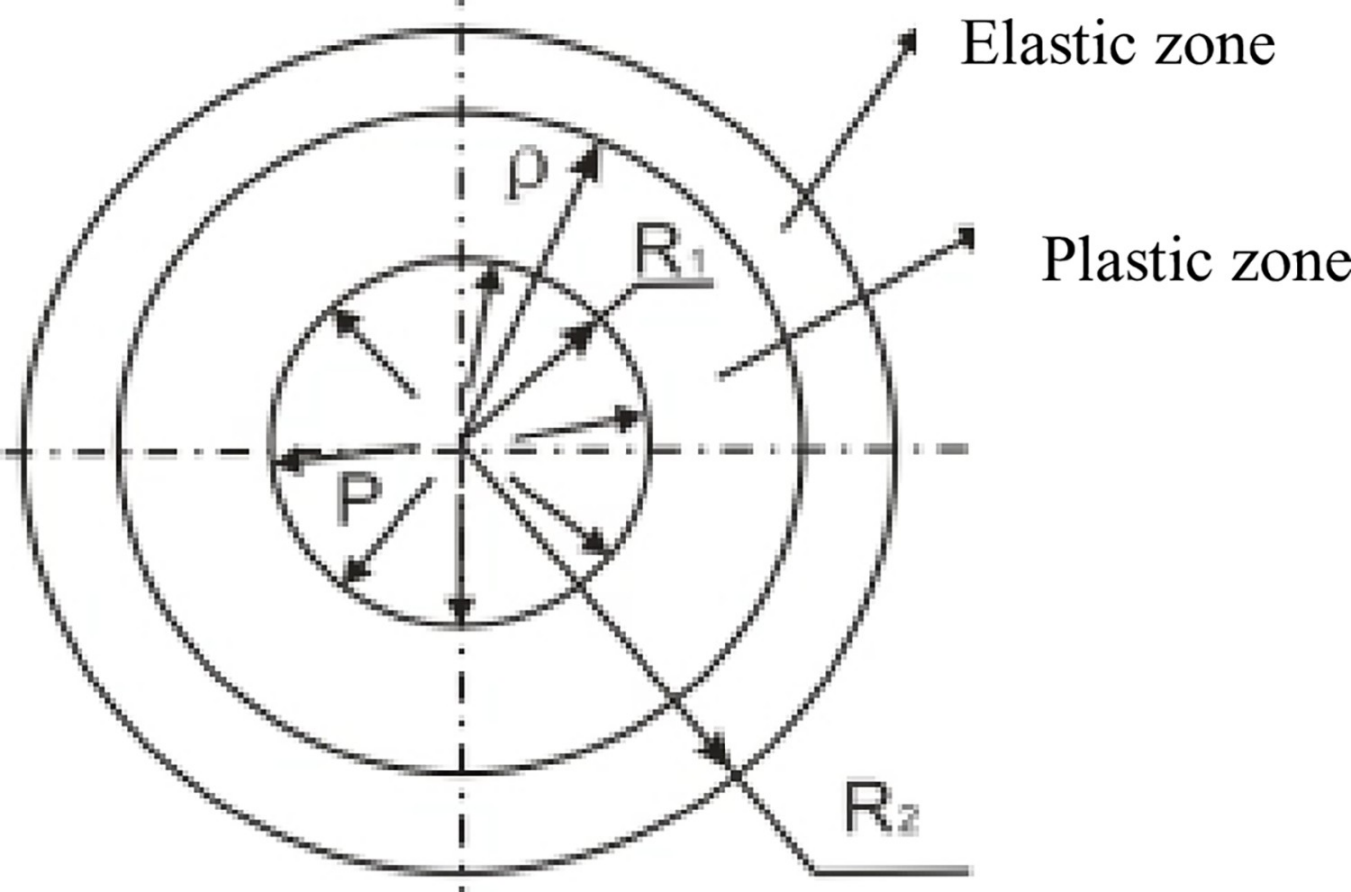
Elastic-plastic stress distribution diagram of thick-walled cylinder.

Due to the simplicity of the constitutive model, requiring only three equations and four parameters, the paper proceeds to demonstrate the application of this constitutive equation. The constitutive equation is employed to analyze the stress distribution around a thick-walled cylinder (Fig 5). The calculation process is as follows, where Eq (4) and Eq (5) represent the shear strain and radial strain, respectively,

$$\varepsilon_{\theta }=\frac{u}{r}$$ (4)

$$\varepsilon_{r}=\frac{du}{dr}$$ (5)

When the elastic modulus *A* is a constant, substituting Eqs (4) and (5) into Eqs (1) and (2), the radial and tangential stress equations are as follows:

$$\sigma_{\theta }=E\times \frac{u}{r}+v\times E\times \frac{du}{dr}$$ (6)

$$\sigma_{r}=E\times \frac{du}{dr}+v\times E\times \frac{u}{r}$$ (7)

$$\frac{\partial \sigma_{r}}{\partial r}+\frac{\sigma_{r}-\sigma_{\theta }}{r}=0\;(equilibrium\;equation)$$ (8)

Substituting Eqs (6) and (7) into Eq (8), we obtain:

$$E\frac{d^{2}u}{dr^{2}}+E\frac{du}{dr}\times \frac{1}{r}-E\frac{u}{r^{2}}=0$$ (9)

From Eq (9), the displacement equation *u* can be calculated, resulting in Eq (10).


$$u=C_{1}\times r+C_{2}\times \frac{1}{r}$$ (10)

At r = r_1_ (within the tunnel) we have:

$$-r_{1}\times p_{1}=r_{1}\times E\times \frac{du}{dr}+r_{1}\times v\times E\times \frac{u_{1}}{r_{1}}$$ (11)

At r = r_2_, (outside the tunnel) we have:

$$-r_{2}\times p_{2}=r_{2}\times A\times \frac{du}{dr}+r_{2}\times v\times A\times \frac{u_{2}}{r_{2}}$$ (12)

Substituting Eq (10) into Eqs (11) and (12), the parameters *C*_1_ and *C*_2_ can be derived,

$$C_{2}=\frac{\left(v+1)(P_{2}-P_{1}\right)}{E({r_{2}}^{2}-{r_{1}}^{2})}\times {r_{2}}^{2}\times {r_{1}}^{2}$$ (13)

$$C_{1}=\frac{\left(1-v)\times (P_{1}{r_{1}}^{2}-P_{2}{r_{2}}^{2}\right)}{E({r_{2}}^{2}-{r_{1}}^{2})}$$ (14)

$$∴\;u=\frac{\left(1-v)\times (P_{1}{r_{1}}^{2}-P_{2}{r_{2}}^{2}\right)}{E({r_{2}}^{2}-{r_{1}}^{2})}\times r+\frac{\left(v+1)(P_{2}-P_{1}\right)}{E({r_{2}}^{2}-{r_{1}}^{2})}\times {r_{2}}^{2}\times {r_{1}}^{2}\times \frac{1}{r}$$ (15)

Substituting Eq (15) into Eqs (4) and (5) respectively, we can obtain the shear strain Eq (16) and the radial strain Eq (17),

$$\varepsilon_{\theta }=\frac{\left(1-v)\times (P_{1}{r_{1}}^{2}-P_{2}{r_{2}}^{2}\right)}{E({r_{2}}^{2}-{r_{1}}^{2})}+\frac{\left(v+1)(P_{2}-P_{1}\right)}{E({r_{2}}^{2}-{r_{1}}^{2})}\times {r_{2}}^{2}\times {r_{1}}^{2}\times \frac{1}{r^{2}}$$ (16)

$$\varepsilon_{r}=\frac{\left(1-v)\times (P_{1}{r_{1}}^{2}-P_{2}{r_{2}}^{2}\right)}{E({r_{2}}^{2}-{r_{1}}^{2})}-\frac{\left(v+1)(P_{2}-P_{1}\right)}{E({r_{2}}^{2}-{r_{1}}^{2})}\times {r_{2}}^{2}\times {r_{1}}^{2}\times \frac{1}{r^{2}}$$ (17)

When *A*(*ε*) varies with strain, the radial and tangential stress equations are as follows:

$$\sigma_{\theta }=E(\varepsilon_{\theta })\varepsilon_{\theta }+vE\varepsilon_{r}$$ (18)

$$\sigma_{r}=E(\varepsilon_{r})\varepsilon_{r}+vE\varepsilon_{\theta }$$ (19)

**Fig 6:**
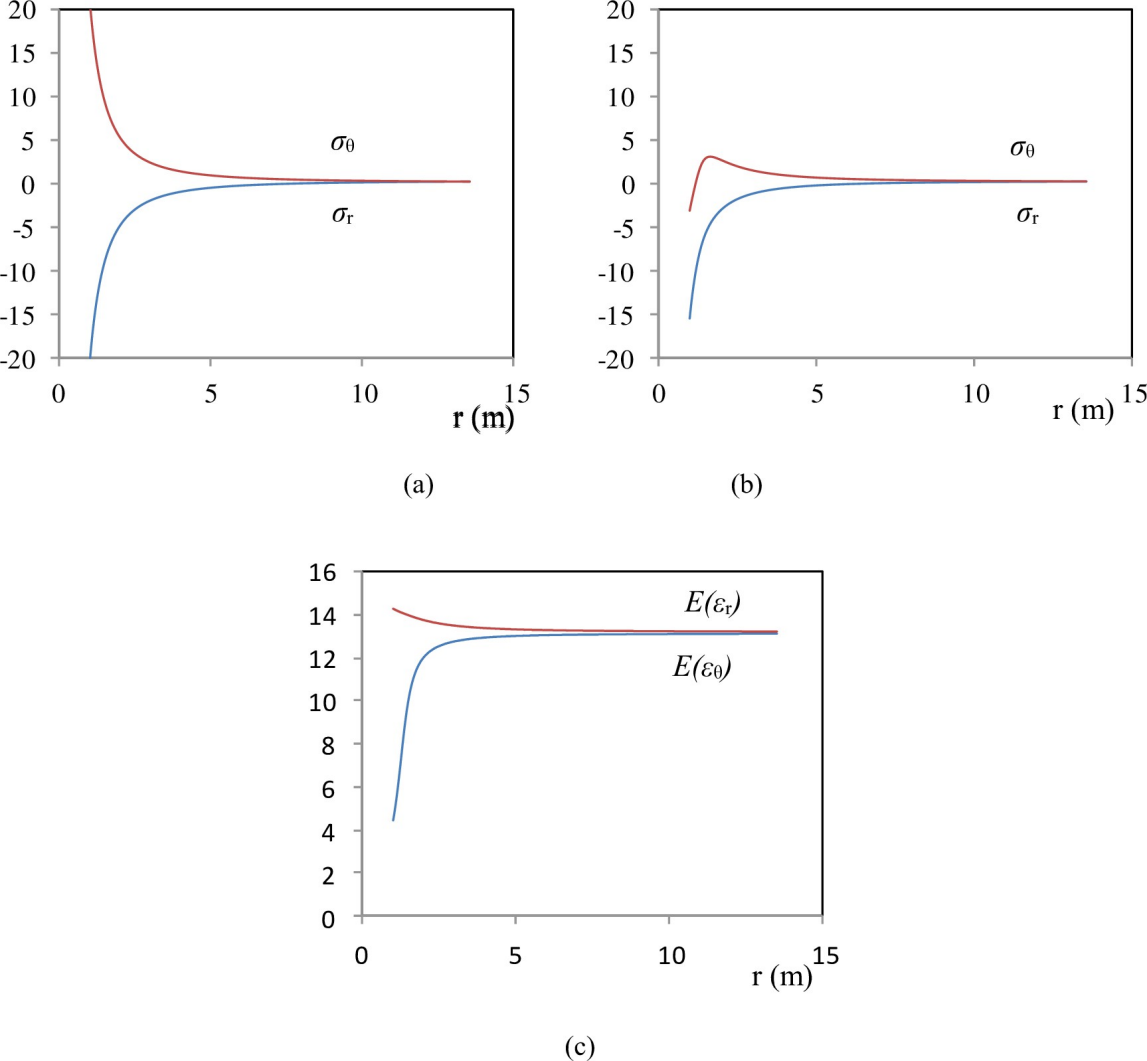
Stress distributions along the cylinder radius (a) as *A*(*ε*) is not const. (b) as *A*(*ε*) is const. (c) *A*(*ε*) distributions along the cylinder radius as *A*(*ε*) is not const. Default values are *r*_1_ = 1 m, *r*_2_ = 10 m, *P*_1_ *=* 20 MPa, *P*_2_ *=* 0 MPa, *E* = 15 GPa, *C =* 1, *ε*_s_ = -0.1, and *v* = 0.1.

By substituting Eqs (16) and (17) into Eqs (18) and (19), the radial stress (*σ*_r_) and tangential stress (*σ*_θ_) distribution around the thick-walled cylinder can be analyzed. The parameters used are *E* = 15 GPa, *v* = 0.1, *C* = 1, and *ε*_s_ = -0.1 (the same as in Fig 3). When *E* is constant, employing Eqs (6) and (7) results in the thick-walled cylinder being in an elastic state (Fig 6A). When E(ε) varies with strain, using Eqs (18) and (19) results in the thick-walled cylinder being in a plastic state (Fig 6B). The primary reason for this is the reduction in the shear modulus in the tangential direction (Fig 6C).

### 3.2. Discussion of results

**Fig 7:**
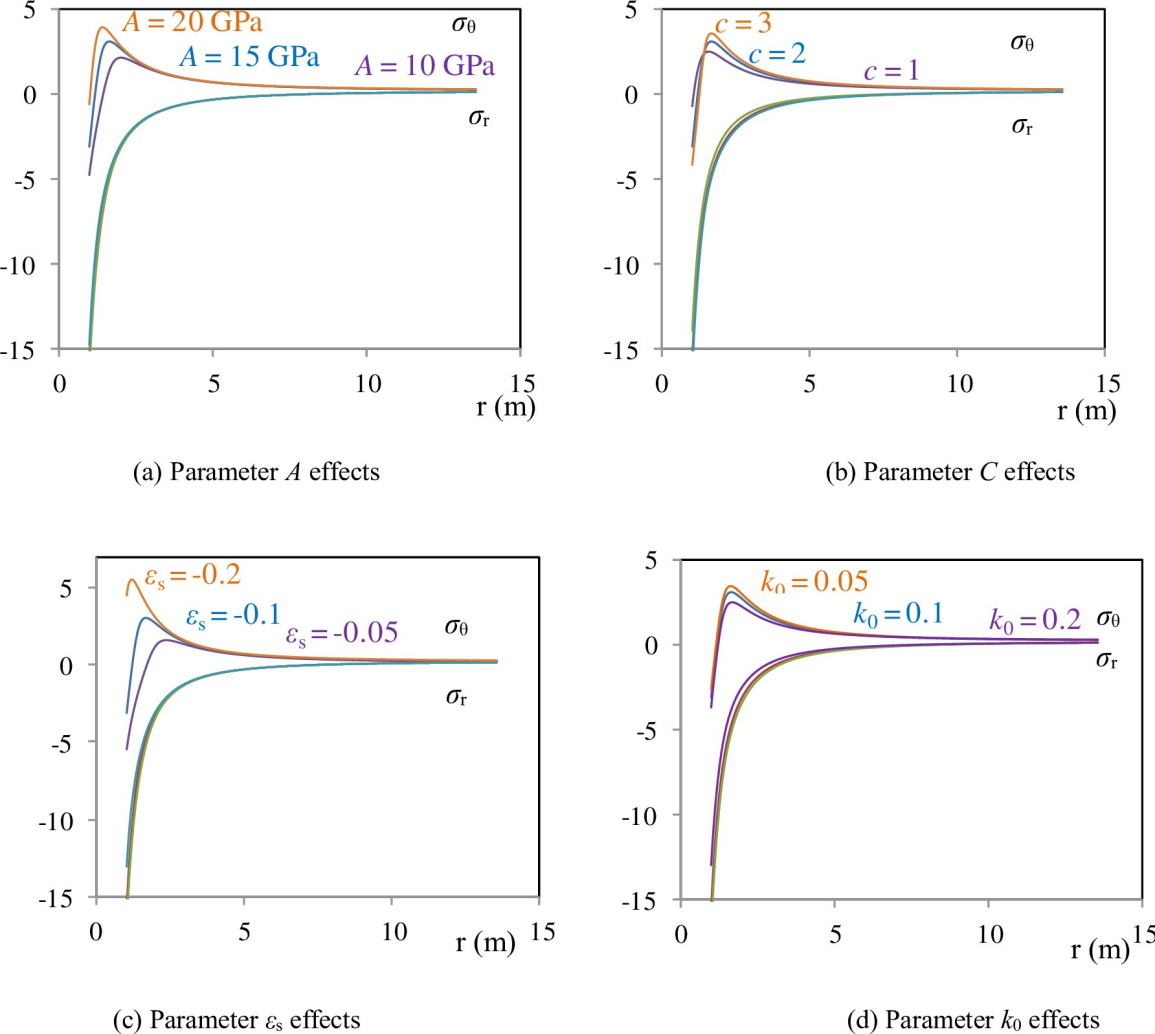
The effects of constants on the stress distributions along the cylinder radius as *A*(*ε*) is not const.

The study investigates the influence of parameters in the constitutive equation on stress distribution. Parameter *A* primarily affects the range of the plastic zone and the magnitude of the maximum tangential stress (Fig 7A); parameter *C* primarily influences the magnitude of the maximum tangential stress (Fig 7B); parameter *ε*_s_ mainly affects the range of the plastic zone and the magnitude of the maximum tangential stress (Fig 7C); parameter v mainly influences the magnitude of the maximum tangential stress (Fig 7D).

## 4. Compared with other constitutive models

**Fig 8:**
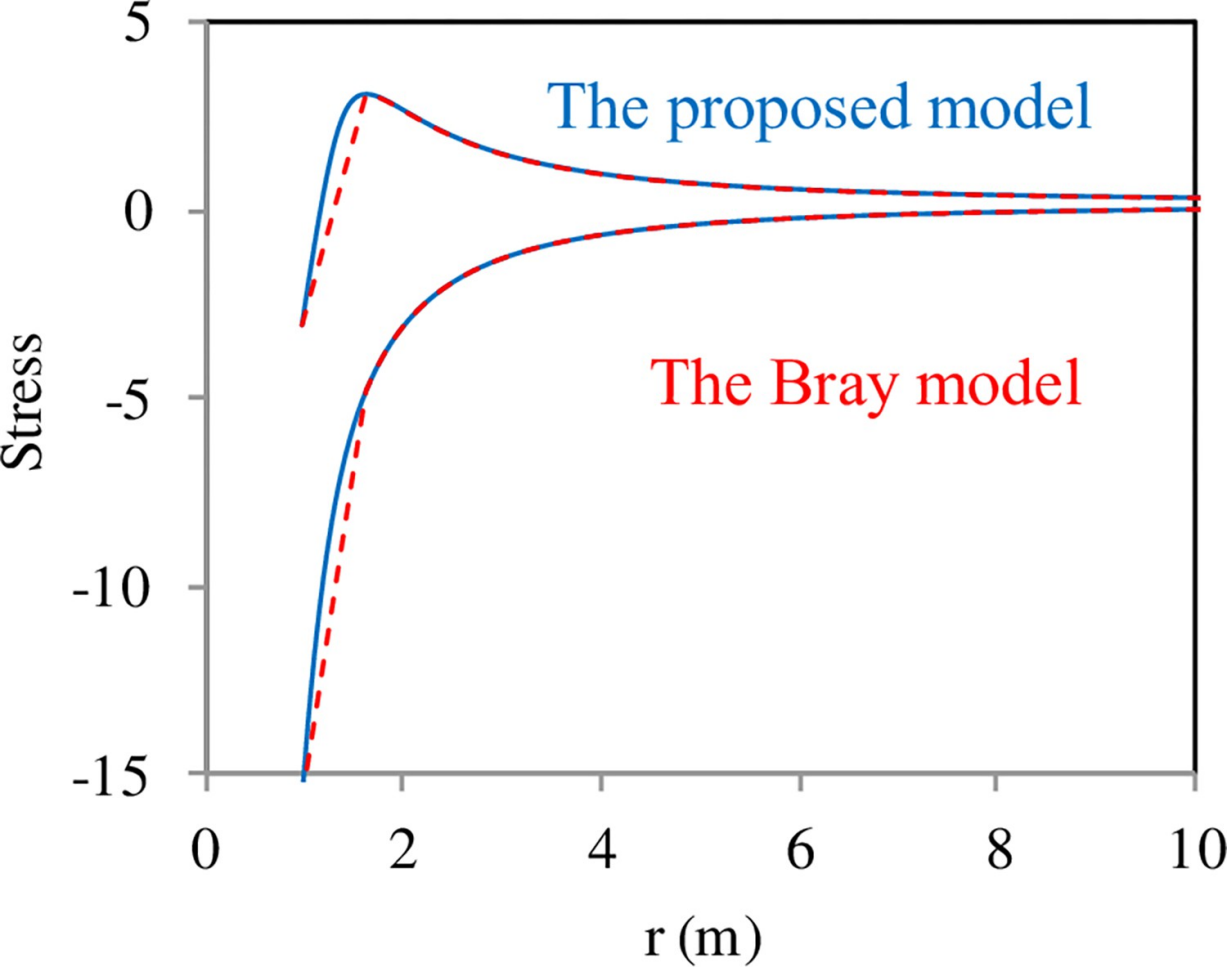
Distribution of stress around the tunnel. The parameters for the proposed model and the Bray model with *r*_1_ = 1 m, *r*_2_ = 10 m, *P*_1_ *=* 20 MPa, *P*_2_ *=* 0 MPa, *E* = 15 GPa, *C =* 1, *ε*_s_ = -0.1, and *v* = 0.1.

The proposed model compared with convertional model (Bray model, Eqs 20–27), and the Distribution of stress around the tunnel has been given in the Fig 8.


$$\sigma_{r}=P_{2}-\frac{b}{r^{2}}$$ (20)

$$\sigma_{\theta }=P_{2}+\frac{b}{r^{2}}$$ (21)

$$b=[\frac{\{\tan^{2}(45+\frac{\varphi }{2})-1\}P_{2}+q_{u}}{\tan^{2}(45+\frac{\varphi }{2})+1}]R^{2}$$ (22)

$$R=r_{1}{\left[\frac{2P_{2}-q_{u}+\{1+\tan^{2}(45+\frac{\varphi_{j}}{2})\}S_{j}\cot\varphi_{j}}{\left\{1+\tan^{2}(45+\frac{\varphi_{j}}{2})\}(P_{1}+S_{j}\cot\varphi_{j}\right)}\right]}^{\frac{1}{Q}}$$ (23)

$$Q=\frac{\tan\delta }{\tan(\delta -\varphi_{j})}-1$$ (24)

$$\delta =45+\frac{\varphi_{j}}{2}$$ (25)

$$\sigma_{r}=(P_{1}+S_{j}\cot\varphi_{j}){\left(\frac{r}{r_{1}}\right)}^{Q}-S_{j}\cot\varphi_{j}$$ (26)

$$\sigma_{\theta }=(P_{1}+S_{j}\cot\varphi_{j})\frac{\tan\delta }{\tan(\delta -\varphi_{j})}{\left(\frac{r}{r_{1}}\right)}^{Q}-S_{j}\cot\varphi_{j}$$ (27)

We got the similar results with Bray model, but distribution of stress around the tunnel are different present that the shape of stress-strain curves are different.

## 5. Conclusions

A constitutive model has been proposed based on the elastic modulus E decreasing with the increase in longitudinal cracks., with the most crucial being the simulation of the stress-strain curve for rocks requiring only three equations (Eqs 12–14) and four parameters (*A*, *k*_0_, *C* and *ε*_*s*_). Therefore, the constitutive equation was utilized to analyze the elastoplastic stress distribution around a thick-walled cylinder. When *A*(*ε*) is constant, the material remains in the elastic region (Fig 5A). In the case where *A*(*ε*) varies with strain, the material enters the plastic region (Fig 5B), primarily due to the reduction in the shear modulus in the tangential direction (Fig 5C). The study further investigated the influence of parameters in the constitutive equation on stress distribution. Parameter *A* primarily affects the range of the plastic zone and the magnitude of the maximum tangential stress (Fig 6A); parameter *C* mainly influences the magnitude of the maximum tangential stress (Fig 6B); parameter *ε*_*s*_ mainly affects the range of the plastic zone and the magnitude of the maximum tangential stress (Fig 6C); parameter *k*_0_ primarily influences the magnitude of the maximum tangential stress (Fig 6D). We got the similar results with Bray model, but distribution of stress around the tunnel are different present that the shape of stress-strain curves are different.

The next step involves incorporating the constitutive model into finite element programs to facilitate a more straightforward and efficient analysis of rock engineering structures. This is expected to contribute to the optimization of practical rock engineering structures and enhance cost-effective design.
